# Supplementary material for: gcplyr: an R package for microbial growth curve data analysis
Source: BMC Bioinformatics. 2024 Jul 9;25:232. doi: 10.1186/s12859-024-05817-3 (PMC11232339; doi:10.1186/s12859-024-05817-3)
Supplement: Supplementary file 1 — Supplementary Material 1. [file 12859_2024_5817_MOESM1_ESM.docx]

**Table S1. gcplyr and other available microbial growth curve data wrangling and analysis tools.**

| **Software** | **Used via** | **Param. analysis** | **Non-param. analysis** | **Scriptable** | **Imports** | **Data reshaping** | **Incorporate designs** | **Plotting** | **Smoothing** | **Citation** |
| --- | --- | --- | --- | --- | --- | --- | --- | --- | --- | --- |
| **gcplyr** | R package | No | Yes | Yes | Block, wide, tidy | Yes, into wide or tidy | Yes | With ggplot2 | Yes | This paper |
| **growthcurver** |  | Yes | Minimally | Yes | Wide | No | No | Built-in | No | [14] |
| **growthrates** |  |  | Minimally | Yes | Tidy | No | No | With ggplot2 | Yes | [15] |
| **biogrowth** |  |  | No | Yes | Wide | No | No | Built-in | No | [16] |
| **opm** |  |  | Minimally | Yes | Specialized formats | Yes, into opm-specific class | Yes | Built-in | Yes | [8] |
| **plater** |  | No | No | Yes | Block | Yes, into tidy | No | No | No | [2] |
| **QurvE** | GUI or R package | Yes | Yes | Yes | Wide, specialized formats, tidy | Yes, from specialized formats into wide | Yes | Built-in | Yes | [3] |
| **Dashing Growth Curves** | GUI | Yes | Minimally | No | Transposed Wide | No | Minimally | Built-in | Yes | [19] |
| **AUDIT** |  | Yes | No | Yes, in part | Tidy, specialized formats | Yes, from specialized formats into tidy | Yes | Built-in, with ggplot2 | Yes | [4] |
| **Parsley** |  | No | No | No | Block, wide | Yes, into tidy | Yes | No | No | [5] |
| **bletl** | Python package | Minimally | Yes | Yes | Specialized formats | Yes | Yes | Built-in | Yes | [6] |
| **AMiGA** | Python package or command line | No | Yes | Some | Wide | No | Yes | Built-in | Yes | [24] |
| **fitderiv** | GUI or Python package | No | Yes | Some | Wide | No | No | Built-in | Yes | [20] |
| **phenom** | Python code | No | Yes | Yes | Wide | No | Yes | No | Yes | [25] |
| **B-GREAT** |  |  | Yes | Yes | Wide | No | Yes | No | Yes | [26] |
| **PMAnalyzer** | bash | Yes | Minimally | No | Wide | No | No | Built-in | No | [17, 18] |
| **GrowthRates** | Command line | No | Yes | No | Wide, specialized formats | Yes | No | No | No | [7] |
| **-----** | MATLAB code | No | Yes | Yes | One well at a time | No | No | No | No | [27] |
| **GCAT** | GUI | Yes | No | No | Wide | No | Yes | Built-in | No | [9] |
| **PRECOG** |  | No | Yes | No | Wide | No | No | Built-in | Yes | [21] |
| **IPMP 2013** |  | Yes | No | No | Wide | No | No | Built-in | No | [10] |
| **GATHODE** |  | No | Yes | No | Wide | No | Minimally | Built-in | Yes | [22] |
| **Microrisk Lab** |  | Yes | No | No | Wide | No | No | Built-in | No | [11] |
| **CarboLogR** |  | Yes | No | No | Specialized formats | No | Yes | Built-in | No | [13] |
| **YODA** | Webpage GUI | No | Yes | No | Wide | No | No | No | No | [23] |
| **BGFit** |  | Yes | No | No | Wide | No | No | Built-in | No | [12] |
